# [Retracted] Progesterone induces cell apoptosis via the CACNA2D3/Ca^2+^/p38 MAPK pathway in endometrial cancer

**DOI:** 10.3892/or.2024.8777

**Published:** 2024-07-15

**Authors:** Xiangnan Kong, Min Li, Kai Shao, Yinrong Yang, Qian Wang, Meijuan Cai

Oncol Rep 43: 121–132, 2020; DOI: 10.3892/or.2019.7396

Following the publication of this paper, it was drawn to the Editor's attention by a concerned reader that certain of the immunohistochemical data shown in Fig. 5C were strikingly similar to data appearing in different form in another article written by different authors at different research institutes that had already been submitted for publication elsewhere prior to the submission of this paper to *Oncology Reports* [Wu X, Cai D, Zhang F, Li M and Wan Q: Long noncoding RNA TUSC7 inhibits cell proliferation, migration and invasion by regulating SOCS4 (SOCS5) expression through targeting miR-616 in endometrial carcinoma. Life Sci 231: 116549, 2019]. In addition, the CACNA203 western blot data shown in Fig. 2A-c and B-C respectively looked strikingly similar, even though different experiments were intended to have been shown in these figure parts. In view of the fact that the contentious data had already apparently been submitted for publication prior to the receipt of this paper at *Oncology Reports*, and owing to a overall lack of confidence in the presentation of the data, the Editor of has decided that this paper should be retracted from the Journal. The authors were asked for an explanation to account for these concerns, but the Editorial Office did not receive a reply. The Editor apologizes to the readership for any inconvenience caused.

